# Supplementary material for: Nitrogen addition enhances seed yield by improving soil enzyme activity and nutrients
Source: PeerJ. 2024 Jan 19;12:e16791. doi: 10.7717/peerj.16791 (PMC10802157; doi:10.7717/peerj.16791)
Supplement: Supplemental Information 1 [file peerj-12-16791-s001.zip › supplementary material/R code.docx]

**Pearson Correlation Code**

library(Hmisc)

df <- read.csv(file.choose(),header = T)

df_rcorr<-rcorr(as.matrix(df) ,type = "pearson")

r <- df_rcorr$r

r

p <- df_rcorr$P

p

write.csv(r,"r.csv")

write.csv(p,"p.csv")

library(dplyr)

library(linkET)

library(ggplot2)

custom_data <- read.csv(file.choose(),header = T,row.names = 1)

env <- read.csv(file.choose(),header = T)

cols <- c(">= 0.05" = "grey", "< 0.05" = "#56B4E9")

qcorrplot(correlate(env), type = "lower", diag = FALSE) +

geom_square() +

geom_couple(aes(colour = coef,linetype= type,size = p_value, from = y, to = x),

data = custom_data, curvature = 0.15) +

scale_fill_gradientn(colours = RColorBrewer::brewer.pal(3, "RdBu")) +

scale_size_manual(values = c(0.5, 1, 2)) +

scale_colour_manual(values = cols) +

scale_linetype_manual(values = c("dashed","solid"))+

guides(size = guide_legend(title = "P value",

override.aes = list(colour = "grey35"),

order = 2),

colour = guide_legend(title = "Coef",

override.aes = list(size = 3),

order = 1),

fill = guide_colorbar(title = "Pearson's r", order = 3))
