# Supplementary material for: Nitrogen addition enhances seed yield by improving soil enzyme activity and nutrients
Source: PeerJ. 2024 Jan 19;12:e16791. doi: 10.7717/peerj.16791 (PMC10802157; doi:10.7717/peerj.16791)
Supplement: Supplemental Information 1 [file peerj-12-16791-s001.zip › supplementary material/Table S1.docx]

Table S1 Physicochemical properties of soil in the test field

| Year | PH | organic matter  /g·kg^-1^ | total nitrogen  /g·kg^-1^ | Alkali-hydrolyzed nitrogen/mg·kg^-1^ | available phosphorus  /mg·kg^-1^ | available potassium  /mg·kg^-1^ |
| --- | --- | --- | --- | --- | --- | --- |
| 2021 | 8.16 | 34.48 | 1.64 | 2.85 | 1.89 | 25.23 |
| 2022 | 8,18 | 33.64 | 1.58 | 2.66 | 1.87 | 24.80 |
