# Supplementary material for: Nitrogen addition enhances seed yield by improving soil enzyme activity and nutrients
Source: PeerJ. 2024 Jan 19;12:e16791. doi: 10.7717/peerj.16791 (PMC10802157; doi:10.7717/peerj.16791)
Supplement: Supplemental Information 1 [file peerj-12-16791-s001.zip › supplementary material/Table S2.docx]

Table S2 abbreviated list

| Abbreviation | Full name |
| --- | --- |
| N | Nitrogen |
| QTP | Qinghai-Tibet Plateau |
| NTs | Number of tillers |
| NFTs | Number of fertile tillers |
| SY | Seed yield |
| pH | Potential of hydrogen |
| SOM | Soil organic matter |
| TN | Total nitrogen |
| TP | Total phosphorus |
| UE | Urease |
| CAT | Catalase |
| SC | Sucrase |
| NR | Nitrate reductase |
