# Supplementary figures and images for: Nitrogen addition enhances seed yield by improving soil enzyme activity and nutrients
Source: PeerJ. 2024 Jan 19;12:e16791. doi: 10.7717/peerj.16791 (PMC10802157; doi:10.7717/peerj.16791)

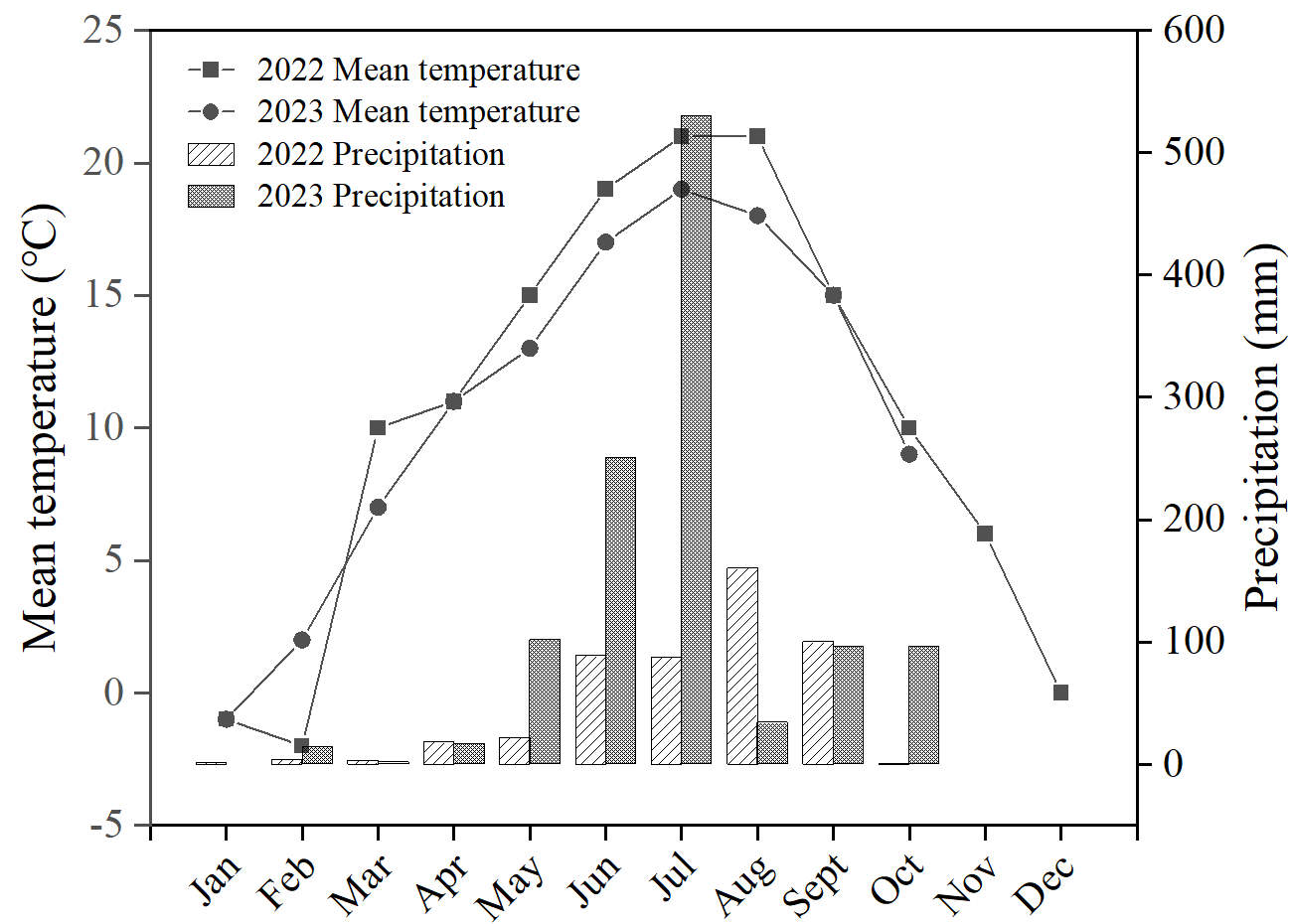

Supplement: Supplemental Information 1 [file peerj-12-16791-s001.zip › supplementary material/Figure S1.png]
